# Alteration of Thyroid Hormone among Patients with Ischemic Stroke visiting a Tertiary Care Hospital: A Descriptive Cross-sectional Study

**DOI:** 10.31729/jnma.6501

**Published:** 2021-08-31

**Authors:** Saroja Bashyal, Manen Prasad Gorkhaly, Rameshwor Devkota, Ramila Devkota, Pradeep Raj Regmi, Isha Amatya

**Affiliations:** 1 Department of Internal Medicine, Rapti Provincial Hospital, Dang, Nepal; 2 Department of Internal Medicine, Bir Hospital, National Academy of Medical Sciences, Kathmandu, Nepal; 3 Deparment of Radiology, Rapti Provincial Hospital, Dang, Nepal; 4 Deparment of Radiology, Bir Hospital, National academy of Medical Sciences, Kathamndu, Nepal; 5 Deparment of Radiology, Hospital for Advanced Medicine and Surgery, Dhumbarahi, Kathmandu, Nepal; 6 Department of Community Medicine, Kathmandu Medical College, Sinamangal, Kathmandu, Nepal

**Keywords:** *hyperthyroidism*, *hypothyroidism*, *ischemic stroke*

## Abstract

**Introduction:**

Stroke is broadly classified as cerebral infarction, intracerebral hemorrhage and subarachnoid hemorrhage. Neuroendocrine profile is altered in acute ischemic stroke and there is a link between hypothyroidism and atherosclerosis which in turn may lead to stroke. The objective of this study was to find out the prevalence of alteration of thyroid hormones in patients with ischemic stroke in a tertiary care center.

**Methods:**

This descriptive cross-sectional study was conducted from June to December 2019 in a tertiary care center. Ethical approval was taken from Institutional review board of National Academy of Medical Sciences (reference number: IM 175). Patients with a diagnosis of stroke, without evidence of cardioembolic source, history of liver disease, renal failure and thyroid disease and who do not use thyroidal supplementation within 180 days prior the event were included. Convenience sampling was done. Data was entered in Microsoft Excel and analyzed using Statistical Package for the Social Sciences version 22. Point estimate at 90% Confidence Interval was calculated along with frequency and percentage for binary data.

**Results:**

The prevalence of altered thyroid levels among 73 patients was 13 (17.8%) (90% Confidence Interval= 10.44-25.16). Among them 11 (15.1%) were hypothyroid and 2 (2.7%) were hyperthyroid. Among severity of hypothyroid cases, subclinical hypothyroidism grade IA was seen in 51 (70%), subclinical hypothyroidism grade IB was seen in 22 (30%),

**Conclusions:**

The prevalence of altered thyroid levels among patients undergoing ischemic stroke was similar to the findings of other international studies.

## INTRODUCTION

Stroke is characterized as a neurological deficit including cerebral infarction, intracerebral hemorrhage (ICH), and subarachnoid hemorrhage (SAH) and is a major cause of disability and death worldwide.^1^ About 1,795,000 people experience a new or recurrent stroke each year.^2^ In developing countries, a majority of the stroke burden is observed, accounting for 75.2% of all stroke-related deaths and 81.0% of the associated DALYs lost.^3^ In Nepal, it was estimated that around 50,000 patients have stroke per year with annual death of around 15000. Of all the ischemic strokes, 77.4% are atherothrombotic occlusion of cerebral blood vessels.^4^

Thyroid dysfunction has been associated with cerebrovascular accidents (CVAs).^5^ Hyperthyroidism may cause ischemic stroke due to its relation to atrial fibrillation (AF).^6^ The main predisposing factors for stroke are hypertension, cigarette smoking, alcohol consumption and diabetes.^4^

The aim of this study was to find out the prevalence of altered thyroid levels among patients with ischemic CVAs in a tertiary care hospital.

## METHODS

This descriptive cross-sectional study was conducted from June to December 2019 in National Academy of Medical Sciences (NAMS), Bir Hospital, Kathmandu. Ethical approval from Institutional review board of NAMS (reference number. IM 175). Convenience sampling was done and the sample size of cases to be enrolled was calculated by using the formula,

n = Z^2^ × p × q / e^2^

  = (1.645)^2^ × (0.5) × (1-0.5) / (0.1)^2^

  = 67.65

Where,

n = minimum required sample size,
Z = 1.645 at 90% Confidence Interval (CI),
p = prevalence taken as 50% for maximum sample size,
q = 1-p
e = margin of error, 10%

Hence, the required sample size was 67.65. Adding a 10% nonresponse rate, we enrolled 73 patients in our study. Patients with clinically diagnosed stroke, presenting within 48 hours of onset of symptoms with CT or MRI findings were included in the study. Patients with prior use of thyroid supplements recently within last 180 days and with a history of liver disease, renal failure and known thyroid disease and who don't give consent were excluded from the study. Informed written consent was taken either in Nepali or in English language, in whichever they felt comfortable. Confidentiality was maintained to the utmost. For those who are not able to give consent because of their clinical state, informed consent was taken from their close relative.

Proforma was translated in Nepali verbally if needed depending upon the cases. For diagnosing the type of stroke, every patient underwent neuroimaging with non-contrast CT of the head. Cerebral infarction was diagnosed if neurological deficits are accompanied by a hypo dense lesion >15 mm in diameter in an appropriate area on a cranial CT scan. Lacunar infarction were diagnosed if the patient presented with a pure motor stroke, a pure sensory stroke, ataxic hemiparesis or a sensorimotor stroke in the absence of a visual field defect and evidence of higher cerebral dysfunction and a hypo dense lesion of < 15 mm in diameter or normal CT scan. In the laboratory, fully automatic analyzer VITROSECi/ECiQ Immunodiagnostic Systems, VITROSTSH was measured using a chemiluminescent immunometric assay with a detection range from 0.01 to 100 m IU/L. TSH values between 0.46 and 4.68 m IU/L were considered the normal reference range (euthyroid). Specimens with TSH values above or below the normal reference range were tested for free thyroxine (FT4). FT4 was measured using a competitive chemiluminescent assay with a detection range of 0.1-12.0 ng/dL. FT4 concentrations within 0.70-2.19 ng/dL was within the normal reference range. All samples included in the study met quality control criteria. Samples with TSH values > 4.69 m IU/L and normal FT4 levels were classified as indicating SCH.

The collected data was stored and analyzed with the Statistical Package for the Social Sciences (SPSS) version 22.0 for windows. Point estimate at 90% Confidence Interval was calculated along with frequency and percentage for binary data.

## RESULTS

The prevalence of altered in thyroid levels among patients undergoing ischemic stroke was 13 (17.8%) and among them 11 (15.1%) were hypothyroid and 2 (2.7%) were hyperthyroid. Minimum age of patient in the study was 26 years and maximum age was 115 years. The mean age of presentation of cerebral infarction in this study was 63.30±19.217.

**Figure 1:**
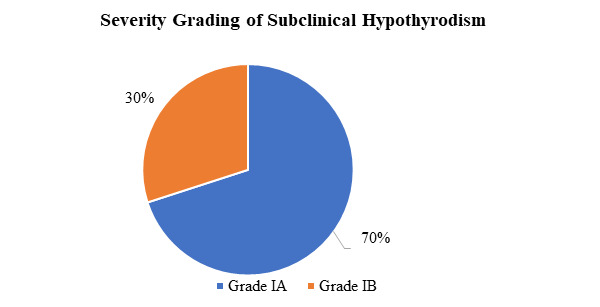
Pie-chart showing severity grading of subclinical hypothyroidism.

Among the total 73 cases, 49 were male and 24 were female. Among 49 males, 37 were euthyroid, 11 were hypothyroid and one male was hyperthyroid. Among 24 females, 23 were euthyroid and one female was hyperthyroid. On further categorization of altered thyroid status, 10 (13.7%) were subclinical hypothyroidism, 1 (1.4%) cases were of overt hypothyroidism and 2 (2.7%) cases had subclinical hyperthyroidism. With regards to the severity of subclinical hypothyroidism, out of the total 10 cases of subclinical hypothyroidism, 70% had grade IA, while the remaining 30% had grade IB severity of the status (Figure 1). In the study 49 (67.12%) of ischemic stroke were male and 24 (32.88%) were female.

Among euthyroid cases mean total cholesterol was 139.55 mg/dl which was higher than the mean cholesterol level in overt hypothyroid cases with 110 mg/dl. But mean total cholesterol level was lower among overt hypothyroid cases compared to euthyroid and overt hypothyroid cases. Mean TC was highest among subclinical hyperthyroid cases. Mean LDLc was highest among euthyroid cases with mean value of 73.94 mg/dl and lowest among overt hypothyroid cases with mean value of 41.00 mg/dl. TG was highest among overt hypothyroid cases with mean value of 199 mg/dl. HDLc was highest among euthyroid cases with mean value of 43.42 mg/dl. Subclinical hypothyroid cases had mean HDL value of 39.90 mg/dl (Table 1).

**Table 1:** Mean value of lipid parameters with thyroid status.

Lipid Parameters	Euthyroid	Subclinical Hypothyroidism	Overt Hypothyroidism	Subclinical Hyperthyroidism
HDLc	43.42	39.90	29.00	36.00
TC	139.55	126.60	110.00	135.00
TG	121.12	120.90	199.00	141.50
LDLc	73.94	65.40	41.00	69.00

## DISCUSSION

Stroke is a form of acute stress with detrimental effect on various neurophysiological pathways.^7^ Hypothyroidism is a possible risk factor for stroke.^8^ A number of comorbidities have been associated with increased mortality in acute stroke patients. It is not known whether hypothyroidism (either clinical or subclinical) affects outcome in patients with acute cerebrovascular disease. A neuroprotective role of hypothyroidism has been shown in acute stroke patients.^8^ Low T3 appeared to be associated with stroke severity and short-term outcome.^9^ In addition, strokes of undetermined etiology accounted for one-third to one-quarter of ischemic strokes among young people and among cases of stroke of undetermined etiology hyperthyroidism could be the underlying cause.^10,11^ So, TFT screening is useful in patients with acute ischemic stroke (IS) because thyroid disorders are known risk factors for cerebrovascular diseases.^6^ In our study we observed 17.8% of the cases had thyroid dysfunction. Our finding is similar to the findings of the retrospective study done by Xu XY , et al. in 893 patients with acute ischemic stroke in which 19% of the cases had abnormal thyroid function tests with at least one of the thyroid related hormones below or above the normal ranges.^12^ Unknown overt or subclinical hyperthyroidism is associated with cardio-embolic stroke.^13^ Thyroid dysfunction in our study was lower than the study done by Dimopolou, et al. in 33 critically ill patients under mechanical ventilation due to acute stroke; study concluded 36% of the cases had thyroid dysfunction.^14^ In our study we found that 1.4% of the cases had overt hypothyroidism, this is comparable to the study done by O'keefe L.M , et al. in 129 patients with acute ischemic stroke. 2.32 % of the cases in the study had hypothyroidism.^15^13.7 % of our study population had subclinical hypothyroidism.

Our result is similar to the study done by Pande, et al. to establish a relation between the variation in thyroid profile and ischemic CVAs in 75 patients within 48 hours of the event, which concluded that 12% of the cases had subclinical hypothyroidism.^7^ Prospective cohort study done by Chakler L, et al. on 47,573 adults (3451 subclinical hypothyroidism) from 17 cohorts concluded an increased risk of stroke on subjects younger than 65 years and those with higher TSH concentrations. Subclinical hypothyroidism (SCH) is postulated to increase stroke risk via atherogenic changes associated with abnormal thyroid function.^16^ The study concluded that hyperthyroidism is associated with an increased risk for ischemic stroke among young adults.^11^

In our study HDLc was low (<40 mg/dl) in all the case with thyroid dysfunction (18% cases) this is much lower compared to the study done by Pokharel , et al., among 281 stroke patients, in which 45% had their HDLc levels below 40 mg/dl.^17^ TG was elevated (>150 mg/dl) in cases with overt hypothyroidism (1.4%) this is consistent with the study done by Abrams JJ , et al. which concluded TG metabolism is not grossly deranged in hypothyroidism.^18^ In our study we found that stroke occurred more commonly among males (67.12%) compared to females (32.88%). Women have lower stroke incidence than men, it might be due to positive effects of estrogen on the cerebral circulation. A lifetime exposure to ovarian estrogens may protect against ischemic stroke.^19^ Seventy-two percent of the cases in our study were smokers. Wang, et al. reported that 48% of patients with stroke smoked.^20^ Tsai, et al. reported 38% of the cases with ischemic stroke were smokers.^21^

Our results might not be generalized to all stroke patients as we recruited study participants from a single hospital only. As many of our patients presented to us after 48 hours of the occurrence of stroke events, we could not involve them in our study.

## CONCLUSIONS

The prevalence of altered in thyroid levels among patients undergoing ischemic stroke was similar to the findings of other international studies. The prevalence of stroke is increasing in developing countries like Nepal as there is rise in non-communicable diseases in this part of world. There are various risk factors for stroke which are modifiable and non-modifiable. So, early identification of these risk factors can have great impact in reduction of morbidity, mortality and burden associated with stroke.

## References

[1] Sacco RL, Kasner SE, Broderick JP, Caplan LR, Connors JJ, Culebras A (2013). An updated definition of stroke for the 21st century: A statement for healthcare professionals from the American heart association/American stroke association.. Stroke..

[2] Benjamin EJ, Muntner P, Alonso A, Bittencourt MS, Callaway CW, Carson AP (2019). Heart Disease and Stroke Statistics-2019 Update: A Report From the American Heart Association.. Circulation..

[3] Venketasubramanian N, Yoon BW, Pandian J, Navarro JC (2017). Stroke Epidemiology in South, East, and South-East Asia: A Review.. J Stroke..

[4] Shaik MM, Loo KW, Gan SH (2012). Burden of stroke in Nepal.. Int J Stroke..

[5] Chung JW, Park SH, Kim N, Kim WJ, Park JH, Ko Y (2014). Trial of ORG 10172 in acute stroke treatment (TOAST) classification and vascular territory of ischemic stroke lesions diagnosed by diffusion-weighted imaging.. J Am Heart Assoc..

[6] Squizzato A, Gerdes VE, Brandjes DP, Buller HR, Stam J (2005). Thyroid diseases and cerebrovascular disease.. Stroke..

[7] Pande A, Goel V, Rastogi A, Gupta A (2017). Thyroid dysfunction in patients of ischemic cerebrovascular accidents.. Thyroid Res Pract..

[8] Alevizaki M, Synetou M, Xynos K, Alevizaki CC, Vemmos KN (2006). Hypothyroidism as a protective factor in acute stroke patients.. Clin Endocrinol (Oxf)..

[9] Zhang Y, Meyer MA (2010). Clinical analysis on alteration of thyroid hormones in the serum of patients with acute ischemic stroke.. Stroke Res Treat.

[10] Rasura M, Spalloni A, Ferrari M, De Castro S, Patella R, Lisi F (2006). A case series of young stroke in Rome.. Eur J Neurol..

[11] Sheu JJ, Kang JH, Lin HC, Lin HC (2010). Hyperthyroidism and risk of ischemic stroke in young adults: a 5-year follow-up study.. Stroke..

[12] Xu XY, Li WY, Hu XY (2016). Alteration of Thyroid-Related Hormones within Normal Ranges and Early Functional Outcomes in Patients with Acute Ischemic Stroke.. Int J Endocrinol..

[13] Bengtsson D, Brudin L, Wanby P, Carlsson M (2012). Previously unknown thyroid dysfunction in patients with acute ischemic stroke.. Acta Neurol Scand..

[14] Dimopoulou I, Kouyialis AT, Orfanos S, Armaganidis A, Tzanela M, Thalassinos N (2005). Endocrine alterations in critically ill patients with stroke during the early recovery period.. Neurocrit Care..

[15] O'Keefe LM, Conway SE, Czap A, Malchoff CD, Benashski S, Fortunato G (2015). Thyroid hormones and functional outcomes after ischemic stroke.. Thyroid Res..

[16] Chaker L, Baumgartner C, Elzen WP, Ikram MA, Blum MR, Collet TH (2015). Subclinical Hypothyroidism and the Risk of Stroke Events and Fatal Stroke: An Individual Participant Data Analysis.. J Clin Endocrinol Metab..

[17] Pokharel BR, Kharel G, Thapa LJ, Rana PV (2015). Vitamin D and Other Risk Factors among Stroke Patients.. Kathmandu Univ Med J (KUMJ)..

[18] Abrams JJ, Grundy SM, Ginsberg H (1981). Metabolism of plasma triglycerides in hypothyroidism and hyperthyroidism in man.. J Lipid Res..

[19] Appelros P, Stegmayr B, Terént A (2009). Sex differences in stroke epidemiology: a systematic review.. Stroke..

[20] China Investigators. (2017). Prevalence, Incidence, and Mortality of Stroke in China: Results from a Nationwide Population-Based Survey of 480 687 Adults.. Circulation..

[21] Tsai CF, Anderson N, Thomas B, Sudlow CL (2015). Risk factors for ischemic stroke and its subtypes in Chinese vs. Caucasians: Systematic review and meta-analysis.. Int J Stroke..

